# Sex Differences in Behavioral Responses to Chronic Unpredictable Mild Stress in Swiss Mice

**DOI:** 10.1111/ejn.70631

**Published:** 2026-07-16

**Authors:** Rachel de Barros‐Telles, Ana Clara F. da Silva, Isabelle Medeiros, Renata V. de Souza, Aline R. Cardoso, Isis N. O. Souza, Gilda A. Neves

**Affiliations:** ^1^ Laboratory of Molecular Pharmacology, Institute of Biomedical Sciences Universidade Federal do Rio de Janeiro Rio de Janeiro Brazil

**Keywords:** anhedonia, anxiety, chronic stress, major depressive disorder, outbred mice, sex differences

## Abstract

Chronic stress‐induced behavioral changes are partly sex‐dependent, but their temporal dynamics remain unclear. Only a few studies have made a direct, comprehensive comparison of sex differences in stress‐induced behavioral responses, and most focus on isogenic mouse strains, overlooking the genetic variability inherent to the stress‐related disorders. In this study, we applied the chronic unpredictable mild stress (CUMS) model in Swiss mice to elucidate the time course of sex‐dependent maladaptive behavioral responses to stress. Adult males and females were exposed to 4, 6, or 8 weeks of CUMS, followed by behavioral assessment and quantification of serum corticosterone. Analysis of the thigmotaxis‐related parameters in the open field (OF) revealed distinct temporal patterns between males and females. CUMS‐exposed females showed an initial decrease in the OF center exploration (4 weeks), with no changes observed after 6 or 8 weeks. By contrast, males showed decreased OF center exploration only after 4 weeks of stress, with this effect shifting to a decrease after 6 and 8 weeks. Eight weeks of CUMS reduced latency and increased immobility during forced swimming. Moreover, only females showed increased corticosterone levels after 8 weeks of stress, and these levels were moderately correlated with forced‐swimming immobility. In conclusion, our results indicate females are more sensitive to stress‐induced maladaptive behavioral responses than males, consistent with clinical data showing that women are more prone to stress‐induced adverse outcomes than men. Moreover, Swiss mice can be considered highly stress‐resilient, requiring longer stress exposure to develop behavioral changes related to depressive‐like symptomatology.

## Graphical abstract

Behavioral changes induced by chronic unpredictable mild stress develop over time in a sex‐dependent manner in Swiss mice. Male and female mice exhibit distinct adaptive strategies to stress in the open‐field exploration profile. Changes in forced‐swimming immobility were robust only after 8 weeks of stress in both sexes. However, only females showed a positive correlation between immobility and serum corticosterone levels, indicating a higher stress sensitivity than males. Swiss mice can be considered a stress‐resilient strain.

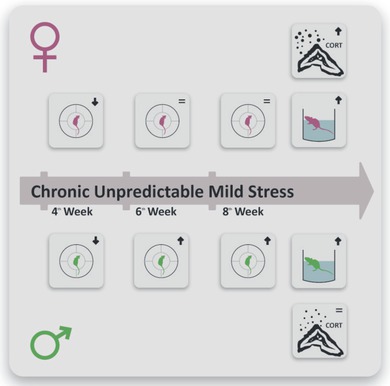

**Abbreviations**
ANOVA: analysis of variance
CRF: corticotropin‐releasing factor
CUMS: chronic unpredictable mild stress
ELISA: enzyme‐linked immunosorbent assay
H: height
HPA: hypothalamus‐pituitary–adrenal
L: length
MDD: major depressive disorder
OF: open field test
PND: post‐natal day
ROUT: robust regression and outlier removal
S.E.M.: standard error of the mean
SPT: sucrose preference test
W: width

## 1 Introduction

Chronic stress is a major risk factor for several neuropsychiatric disorders, including mood and anxiety‐linked ones (Smoller 2016). Major depressive disorder (MDD) affects over 320 million people globally, is currently the 13th major cause of disability, and is expected to be the leading cause by 2030 (Global Burden of Disease Collaborative Network 2024). MDD can cause emotional, cognitive, and social impairment in affected individuals (Malhi and Mann 2018), and its complex etiology makes it challenging to establish an effective pharmacotherapy, with approximately one‐third of MDD patients remaining symptomatic (Rush et al. 2006). Symptoms may include depressive mood, anhedonia, weight and appetite changes, sleep disorders, and reduced focus on activities (American Psychiatric Association 2013). MDD often co‐occurs with anxiety disorders (Kessler et al. 2015), which affect around 460 million people worldwide (Wang et al. 2025). Together, these mental disorders make up the most prevalent psychiatric disorders, imposing a global annual loss of more than a trillion dollars in productivity (Chisholm et al. 2016).

The role of chronic stress in the onset and progression of depression and anxiety is partially explained through the several brain changes it can promote, including decreased neurogenesis (Schoenfeld et al. 2017) and atrophy of the cortical and limbic regions (Marsden 2013). It also triggers anxiety‐ and depression‐like behavioral changes in rodents (Willner 2017). Additionally, long‐term exposure to environmental stressors can lead to dysfunction in the hypothalamus‐pituitary–adrenal (HPA) axis, including its hyperactivity, impaired negative feedback mechanisms, and increased corticotropin‐releasing factor (CRF) expression, resulting in elevated systemic glucocorticoid levels (Palumbo et al. 2020).

Importantly, stress‐induced changes in the brain and behavior are influenced by sex (McEwen and Milner 2007; Ter Horst et al. 2009; Bangasser and Cuarenta 2021). In agreement, depression is 50% more frequent in women than in men (Global Burden of Disease Collaborative Network 2024), and women with MDD have higher rates of comorbidity with anxiety disorders (Kessler et al. 2015). Some preclinical studies have shown that females not only release more CRF in response to stress than males but also exhibit less negative feedback by corticosterone, leading to higher glucocorticoid levels (Bangasser and Valentino 2014). Nevertheless, differences in the time course of stress‐induced behavioral changes between males and females, including neuroendocrine changes, remain poorly characterized, partly because of the lack of preclinical studies in females.

The chronic unpredictable mild stress (CUMS) model is a well‐established rodent model for exploring the relationship between stress exposure, changes in the neuroendocrine pathways, and behavioral changes (Willner 2017). CUMS allows the observation of behavioral parameters such as cognitive impairment, anxiety, and measures of passive stress coping related to depression (Elizalde et al. 2008; Antoniuk et al. 2019), being a valuable tool for characterizing sex differences in stress response. However, only a few studies have made a direct, comprehensive comparison of sex differences in stress‐induced behavioral responses in mice. Moreover, most studies focus on isogenic genetically identical, inbred, mouse strains such as BALB/c and C57BL/6 (Antoniuk et al. 2019), focusing on overstated possible lower data variability (Tuttle et al. 2018), and overlooking the genetic variability inherent to the stress‐related disorders (Moore et al. 2020). By using Swiss mice, an outbred, genetically diverse strain, we can better model the genetic heterogeneity seen in humans, thereby maximizing the robustness of conclusions drawn from environmental perturbations such as CUMS (Saul et al. 2019). Thus, this study aimed to investigate sex differences in the time course of stress‐induced changes in Swiss mice by varying the duration of stress exposure, assessing behavior related to anxiety and depressive‐like symptomatology across multiple behavioral tasks.

## 2 Materials and Methods

### 2.1 Animals

All animal experiments were conducted in accordance with the ARRIVE guidelines, the Council Directive 2010/63/EU of the European Parliament, and the Brazilian National Council for Animal Experimentation Control (CONCEA) ethical guidelines (CEUA/CCS/UFRJ, approval number 100/19). Adult (PND > 60) female (*n* = 76) and male (*n* = 72) Swiss mice from the Institute of Biomedical Sciences of the Federal University of Rio de Janeiro breeding colony were used. Mice were housed in groups of 3–5 per cage. All animals were kept at 23°C ± 2°C on a 12‐h light–dark cycle (lights on at 6 a.m.). Body weight was monitored weekly.

### 2.2 CUMS

Animals were assigned to CUMS or control groups using a block‐randomized design, where all animals in the same cage were assigned to the same group. This approach is fundamental, since a stressed animal in a cage can stress all its cagemates. Three different animal cohorts were used: (1) 4 weeks of stress exposure (males: *n* = 12 control and 12 CUMS; females: *n* = 12 control and 16 CUMS), (2) 6 weeks of stress exposure (males: *n* = 12 control and 12 CUMS; females: *n* = 12 control and 12 CUMS), and (3) 8 weeks of stress exposure (males: *n* = 12 control and 12 CUMS; females: *n* = 12 control and 12 CUMS). Cages were allocated in specific cohorts (stress exposure duration) by simple randomization. The respective control groups were kept in the animal facility for the same amount of time and were disturbed only for routine procedures.

CUMS groups were randomly exposed to a series of environmental stressors (five stressors per week, 5 days a week), including strobe light, white noise (90 dB), overnight food deprivation, overnight water deprivation, 2‐h restraint, overnight lighting, predator odor exposure, cage tilting (45°), overnight wet bedding material, or overnight no bedding material. Stressors were presented in a randomized order, and the selected stressors were changed weekly. Moreover, occasionally, some stressors were applied simultaneously. All overnight procedures lasted 12–16 h. An example of a four‐week protocol is shown in Table 1.

**TABLE 1:** Chronic unpredictable mild stress (CUMS) protocol. Example of four weeks of randomized stressors.

Week	Sunday	Monday	Tuesday	Wednesday	Thursday	Friday	Saturday
1st		No bedding material overnight	Food deprivation overnight	White noise exposure (5 h)	Cage tilting + strobe light (5 h)	Predator odor exposure (2 h)	
2nd		Water deprivation overnight	Cage tilting + white noise (5 h)	Overnight lighting	Restraint (2 h)	No bedding material overnight	
3rd		Food deprivation overnight	Cage tilting (5 h)	Predator odor exposure (2 h)	Wet bedding material overnight	Strobe light + white noise (5 h)	
4th		Cage tilting (5 h)	Water deprivation overnight	Strobe light (5 h)	Overnight lighting	Restraint (2 h)	

In order to discard any possible effect of stress on female cycling, the estrous cycle was monitored at baseline and then weekly by vaginal wash in both control and CUMS exposed groups. Every stage of the estrous cycle was identified in both groups, regardless of stress duration, arguing in favor of continuous cycling throughout the study. The complete methodology and observed results are available in Table S1.

### 2.3 Behavioral Experiments

Behavioral experiments were conducted during the animals' light cycle in a room with indirect, dim lighting (approximately 20 lx). Animals were habituated to the experimental room for 1 h before the experiments. Males and females were tested separately. All experiments were conducted and analyzed by experimenters blinded to the previous intervention.

#### 2.3.1 Sucrose Preference Test (SPT)

The SPT employed was based on pilot experiments from our group and literature data (Schall et al. 2020; Belloch et al. 2021; Meade et al. 2021). All animals were habituated to a 2.5% sucrose solution for 48 h and deprived of access to any liquids for 16 h prior to the experiment. To assess anhedonic behavior, each animal was individually placed in a testing chamber with access to two identical 250 mL plastic sipper tube bottles, one containing filtered water and the other containing a 2.5% sucrose solution, for 30 min. The positions of the bottles were switched after 15 min to minimize side‐preference bias. At the end of the procedure, the bottles were weighed, and the percentage of sucrose consumption was calculated from the total liquid consumed. Sucrose preference was assessed at baseline (prior to stress exposure) and 24 h after the last stressor presentation. No exclusion criterion based on baseline sucrose preference was established.

#### 2.3.2 Social Approach Test

Animals were placed in an acrylic box (60 cm L × 45 cm W × 30 cm H) divided into three equal chambers (20 cm L × 45 cm W each), separated by two removable dividers. The lateral chambers had two identical aluminum cages (9.5 cm H, 8 cm in diameter) to contain the social or neutral stimulus (object). The experiment was conducted as previously described (Gonçalves et al. 2023). Briefly, the social stimulus consisted of an unfamiliar, same‐sex younger mouse. On the first day, each test mouse was habituated for 5 min in the central chamber. Subsequently, the dividers were removed, and animals were allowed to freely explore all three chambers for 10 min. No stimuli were included inside the cages. On the second day, the same protocol was repeated with the addition of the stimuli. In one lateral chamber, an object was placed (neutral stimulus), while the other chamber contained a social stimulus. The test was videotaped, and cage exploration was manually timed. Cage exploration was defined as sniffing, touching, or poking the cages. The percentage of time spent exploring the social and the neutral cages was calculated.

#### 2.3.3 Open Field (OF) Test

Animals were placed in the center of the OF for 6 min to record their locomotor activity and assess indicators of anxiety‐like behavior. The OF consisted of a circular arena (30 cm H, 40 cm in diameter), virtually divided into a central circular area (20 cm in diameter) and a peripheral area (10 cm wide). The experiment was recorded, and the total displacement (cm), as well as the time spent (s) and distance travelled (%) in the center of the apparatus were determined using the MouseGlob software (Borsoi et al. 2021).

#### 2.3.4 Forced Swimming Test

Animals were individually placed inside a cylinder (30 cm H, 10 cm in diameter), containing a 15 cm column of water at 24°C ± 1°C, where they swam for 6 min. To assess stress coping behavior, the animals' latency to the first immobility period lasting more than 1 s (Castagné et al. 2011) and their total immobility time were analyzed. Mice were considered immobile when they made only the minimum movements necessary to keep their heads above water. This test was performed immediately after the OF, as previously described (Borsoi et al. 2021).

### 2.4 Corticosterone Assay

#### 2.4.1 Blood Sampling

Blood samples were collected between 49 and 54 h after the last behavioral experiment, in order to reflect basal corticosterone levels rather than an acute increase induced by FST. Briefly, animals were anesthetized using isoflurane (3%) and decapitated during the light cycle, between 1 pm and 3 pm, to avoid the afternoon rise of corticosterone levels (Gong et al. 2015). Whole blood from the body trunk was collected and placed on ice. Samples were immediately centrifuged at 12,000 rpm for 10 min, and the supernatants (serum) were collected and stored at −80°C until analysis.

#### 2.4.2 Corticosterone ELISA Assay

Corticosterone plasma levels were measured using a commercial ELISA kit (No. 501320, Cayman Chemical Company, USA) according to the manufacturer's instructions (assay range: 8.2–5000 pg/mL; limit of quantification: 30 pg/mL, intra‐assay variability: 4.9%–9.3%, and inter‐assay variability: 8.8%–9.3%) (Cayman Chemical 2021). Briefly, corticosterone was extracted from 100 μL of serum samples by adding 4 × 400 μL of dichloromethane. After mixing, the organic phase was collected. The solvent was removed under nitrogen, and the solid residue was dissolved in 1.4 mL of ELISA buffer. Samples were further diluted (1:20) prior to assay. Fifty microliters of each sample were added to the plate wells and incubated overnight at 4°C with 50 μL of corticosterone acetylcholinesterase (AChE) tracer and 50 μL of the ELISA antiserum. After washing, the reaction was developed by adding 200 μL of Ellman's reagent. Detection was performed using a spectrophotometer at 412 nm for 2 h.

### 2.5 Statistical Analysis

Sample size was previously calculated using G*Power v. 3.1.9.7, based on published data from our group using the same behavioral endpoints (Borsoi et al. 2021). Considering a moderate effect size (0.26) and standard values for α (0.05) and power (0.80), the total sample size was estimated as 146 (12–13 animals per group). For corticosterone determination, a larger effect size (0.55) was expected, leading to a sample size of 40 (5 animals per group). The choice of hypothesis test was based on the research group's expertise in the analyzed outcomes and the current recommendation of the local ethical committee on animal research (CEUA/CCS/UFRJ). GraphPad Prism v. 8.0.2 (GraphPad), SigmaStat v. 11 (Jandel Scientific Corporation), and SPSS Statistics v. 24.0 (IBM) were used for data analysis. The normality of the data was assessed using the Shapiro–Wilk normality test, and the homogeneity of variances was assessed using the Levene median test. Outlier data points were identified using the robust regression and outlier removal (ROUT) method, with a 1% false‐positive threshold, and were excluded from the posterior analysis. One‐sample Student's *t*‐tests were applied to compare the results with the theoretical means of 50% in the SPT and social approach tests. For all behavioral outcomes, a three‐way analysis of variance (ANOVA) was used to compare data between experimental groups, using sex (male or female), intervention (control or CUMS), and stress duration (4, 6, and 8 weeks) as independent factors. For weight‐gain data, a three‐way ANOVA with repeated measures was conducted, with intervention and sex as independent factors and time (weeks) as the repeated factor. Tukey's post hoc test was used to follow up on all ANOVAs. A *p*‐value < 0.05 indicated statistical significance. Data are expressed as the mean ± standard error of the mean (S.E.M.). The complete description of the statistical analysis results (Tables S2–S7) is available in the Supporting Information file.

## 3 Results

### 3.1 Weight Gain

**FIGURE 1:**
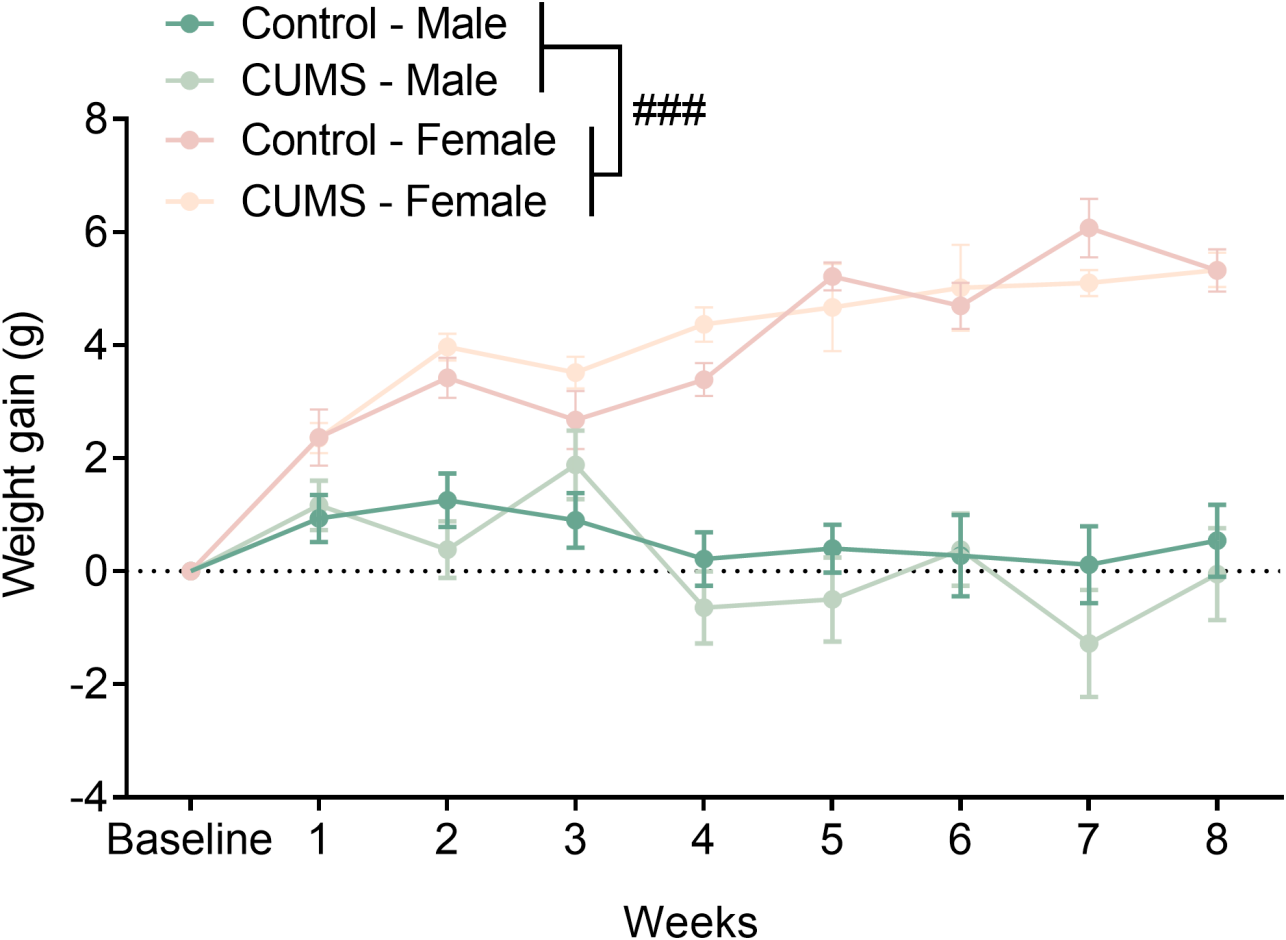
CUMS exposure did not affect mice's weight gain. Three‐way repeated measures ANOVA, sex factor, ^###^ *p* < 0.001 (*n* = 12).

One of the known outcomes of chronic stress exposure is changes in eating behavior and, consequently, weight gain. Our CUMS experimental protocol did not affect mice's weight gain throughout the weeks (intervention factor, F_(1,44)_ = 0.326, *p* = 0.571, Figure 1). However, a significant sex and time interaction (F_(8,352)_ = 17.087, *p* < 0.001) was observed. While female mice showed a significant increase in body mass in all weeks (*p* < 0.001 all weeks vs. baseline in the post hoc analysis), males' weight did not significantly change compared to baseline (*p* > 0.103 all weeks vs. baseline in the post hoc analysis).

### 3.2 Stress Exposure Did Not Induce Anhedonia or Social Impairment in Swiss Mice

**FIGURE 2:**
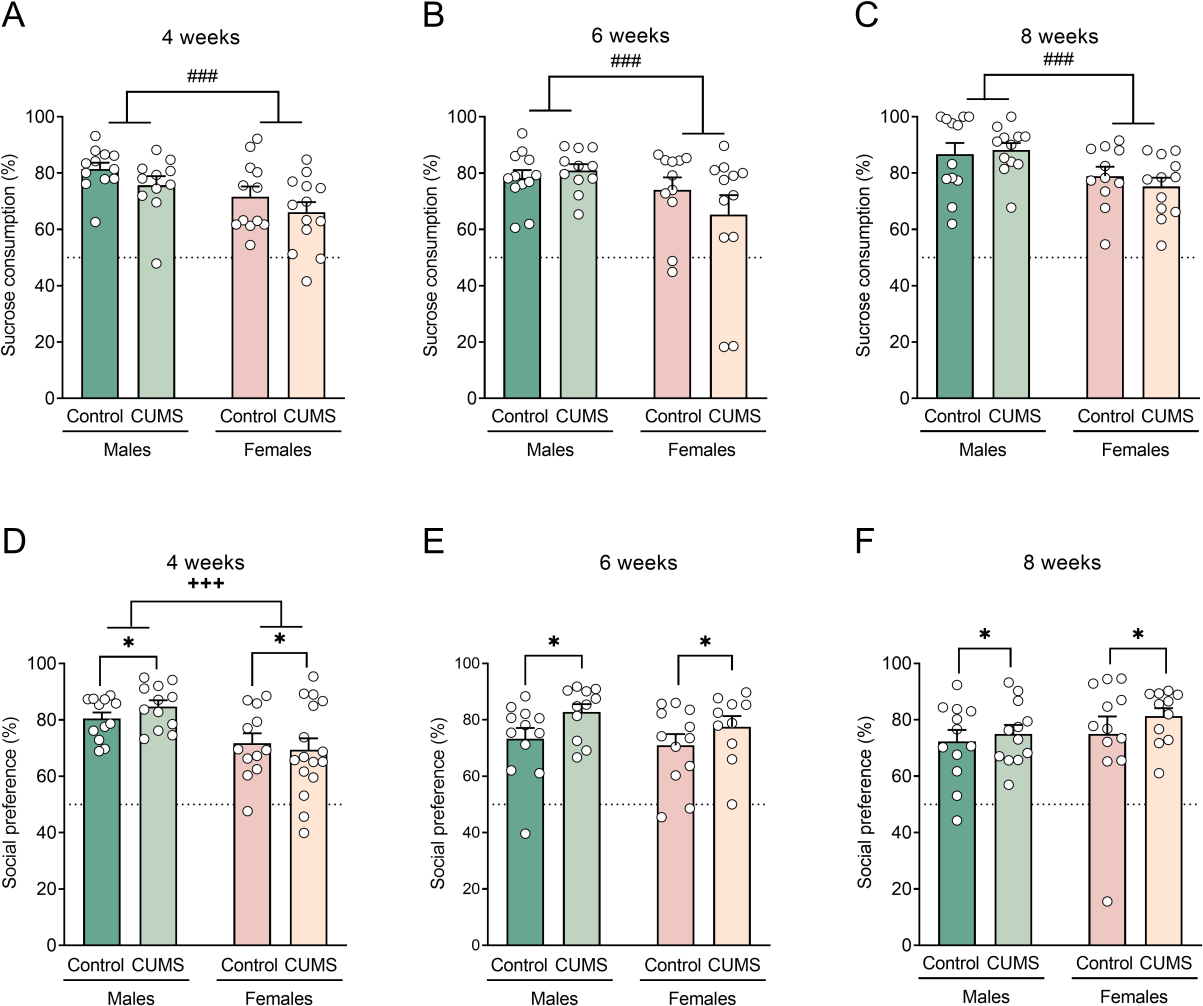
CUMS exposure did not affect sucrose and social preference in mice. (A–C) Sucrose preference (%) after 4, 6, and 8 weeks of stress exposure (*n* = 11–13). Three‐way ANOVA, sex factor, ^###^ *p* < 0.001. (D–F) Interaction time (%) with the social cage during the social approach test after 4, 6, and 8 weeks of stress exposure (*n* = 10–16). Three‐way ANOVA, intervention factor, *p < 0.05; sex vs. stress duration interaction, ^**+++**^ *p* < 0.001.

The SPT is a classical method for assessing anhedonia in animal models of stress‐related disorders (Primo et al. 2023). In our study, baseline sucrose preference was similar between males and females (Figure S1), and all groups presented a significant preference for sucrose consumption over water (*p* < 0.001). After stress exposure (Figure 2A–C), all experimental groups exhibited sucrose consumption above the theoretical 50% threshold, showing a significant preference for sucrose over water (one‐sample Student's *t*‐test vs. 50%, Table S3). Thus, we were unable to observe the development of anhedonia following stress exposure. However, male mice showed greater sucrose preference than females in all cohorts (sex factor, F_(1,129)_ = 21.658, *p* < 0.001), regardless of stress exposure. These data show a sex difference related to hedonic responses in Swiss mice.

In the social approach test (Figure 2D–F), CUMS exposure induced a small increase in social preference regardless of stress duration and sex (intervention factor, F_(1,132)_ = 4.206, *p* = 0.042). However, all groups, independently of stress exposure, exhibit a robust social preference (one‐sample Student's *t*‐test vs. 50%, Table S3), with average interaction values above 70. Considering sex differences, a significant sex vs. stress duration interaction was detected (F_(2,132)_ = 4.887, *p* = 0.009), indicating that female mice showed reduced social preference in the 4‐week groups (*p* < 0.001). Nonetheless, this observation was not reproduced in the 6‐week (*p* = 0.330) or 8‐week (*p* = 0.241) groups.

### 3.3 CUMS Exposure Induced Distinct Effects in Anxiety‐Like Behaviors in Males and Females

**FIGURE 3:**
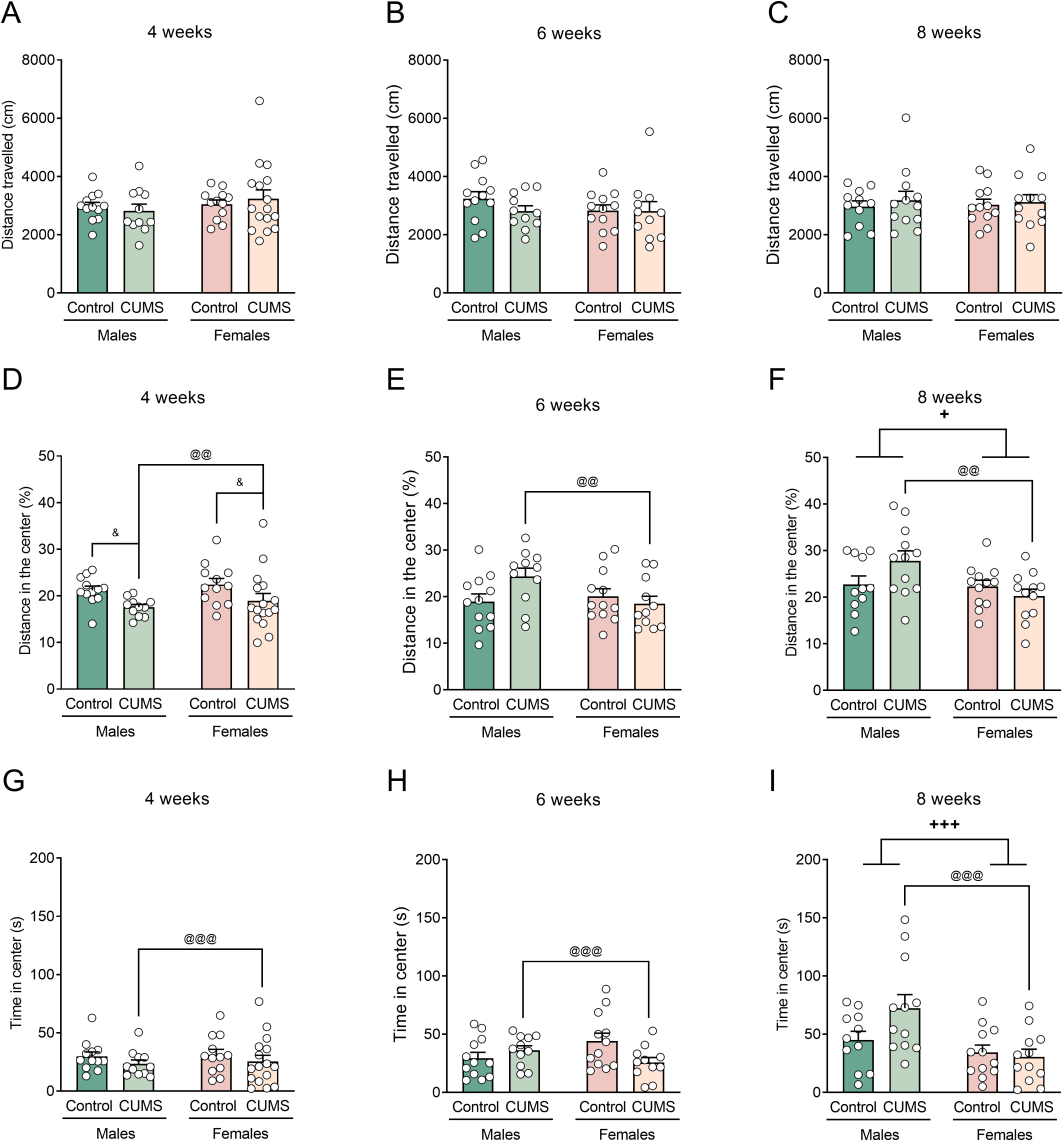
CUMS induced a sex‐specific effect in the anxiety‐related parameters evaluated in the open field (OF) test (*n* = 11–16). (A–C) Total distance travelled (cm) after 4, 6, or 8 weeks of stress exposure. (D–F) Distance travelled (%) in the center of the OF after 4, 6, or 8 weeks of stress exposure. Three‐way ANOVA, sex vs. intervention interaction, ^@@^ *p* < 0.01; sex vs. stress duration interaction, +*p* < 0.05, intervention vs. stress duration interaction, ^&^ *p* < 0.05. (G–I) Time spent (s) in the center of the OF after 4, 6, or 8 weeks of stress exposure. Three‐way ANOVA, sex vs. intervention interaction, ^@@@^ *p* < 0.001; sex vs. stress duration interaction, ^**+++**^ *p* < 0.001.

Evaluating the distance travelled by mice in the OF, no significant differences were detected between experimental groups (Figure 3A–C). Thus, stress exposure did not affect the mice's motor behavior. During this same task, some parameters related to anxiety were analyzed, including the percentage of distance travelled (Figure 3D,E) and time spent (Figure 3G,H) in the center of the apparatus. After 4 weeks of stress exposure, animals subjected to CUMS exhibited reduced locomotion in the field center (intervention vs. stress duration interaction, F_(2,132)_ = 3.950, *p* = 0.022, 4‐week cohort post hoc *p* = 0.018), unrelated to sex (Figure 3C). This can be interpreted as a stress‐induced increase in anxiety. However, it was not accompanied by a reduction in time spent at the OF center (intervention vs. stress duration interaction, F_(2,132)_ = 1.930, *p* = 0.149).

**FIGURE 4:**
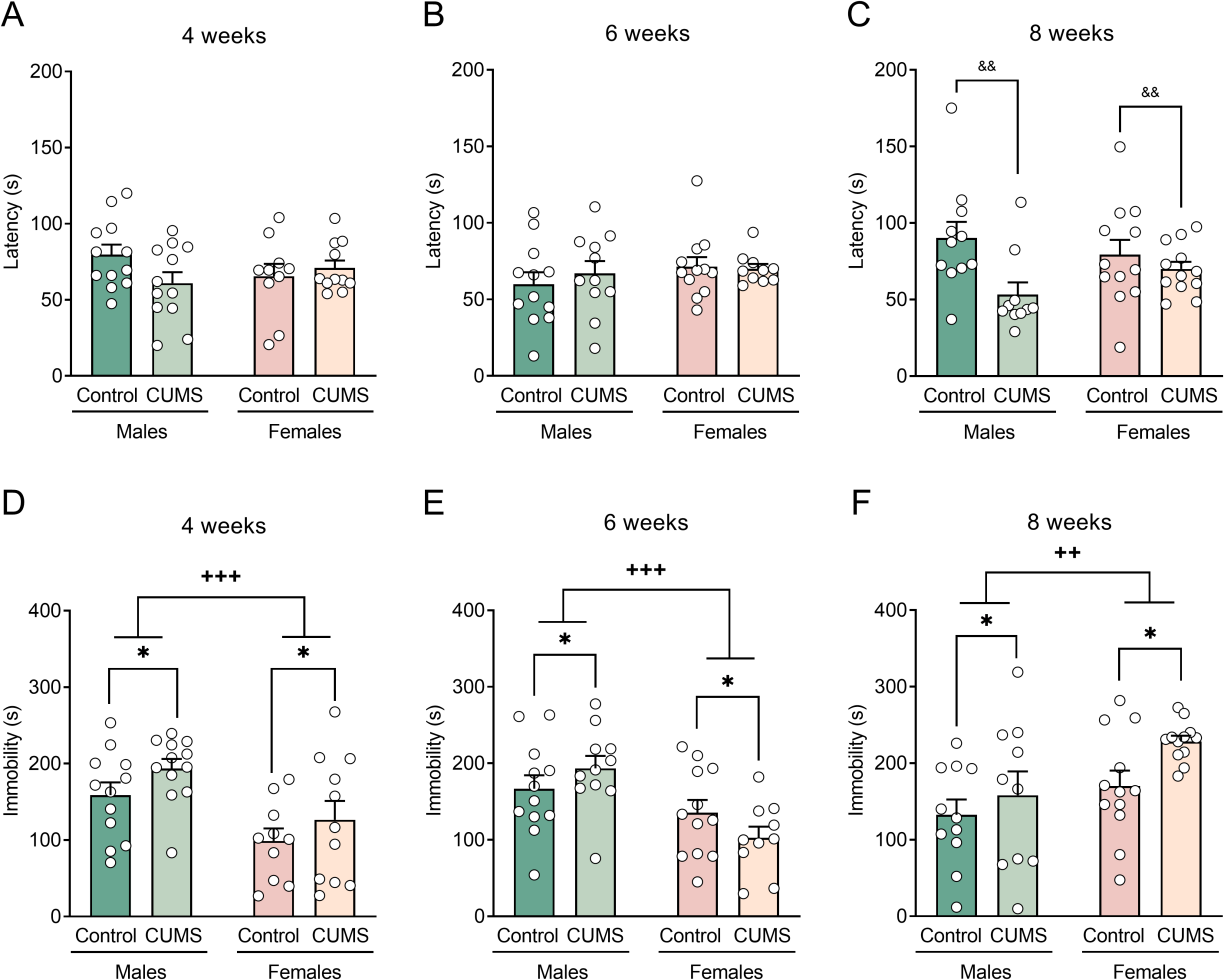
CUMS reduced latency and increased immobility duration after 8 weeks of exposure (*n* = 10–12). (A–C) Latency (s) to immobility after 4, 6, or 8 weeks of stress exposure. Three‐way ANOVA, intervention vs. stress duration interaction, ^&^ *p* < 0.05. (D–F) Immobility time (s) during the forced swimming test after 4, 6, or 8 weeks of stress exposure. Three‐way ANOVA, intervention factor, *p < 0.05, sex vs. stress duration interaction ^**++**^ *p* < 0.01, ^**+++**^ *p* < 0.001.

In addition, a significant interaction between sex and stress intervention was detected in both distance (F_(1,132)_ = 6.789, *p* = 0.010) and time (F_(1,132)_ = 6.552, *p* = 0.012) in the OF center. Especially after 6 and 8 weeks of stress exposure (Figure 4D,E,G,H), males exposed to CUMS showed increased field center exploration, as evidenced by a significantly greater percentage of distance travelled (*p* = 0.001) and time spent (*p* < 0.001) than CUMS‐exposed females. These data indicate that while stress‐exposed females seemed to adapt to the anxiogenic effects of stress, stressed males showed an altered stress coping response. Finally, the sex vs. stress duration interaction analysis revealed a significant sex difference in field exploration (distance: F_(2,132)_ = 3.114, *p* = 0.048; time: F_(2,132)_ = 6.783, *p* = 0.002) that became significant after 8 weeks of the protocol (Figure 4F,I). Males spent significantly more time (*p* < 0.001) and travelled a greater distance (*p* = 0.018) in the field center than females, regardless of stress exposure.

### 3.4 Mice Exposed to 8 Weeks of CUMS Showed Decreased Latency and Increased Forced Swimming Immobility

The immobility behavior during the forced swimming test is considered a stress‐coping response that can be influenced by previous exposure to stressful situations (Borsoi et al. 2021). In this test, latency to the first immobility bounce analysis showed a significant intervention vs. stress duration interaction (F_(2,123)_ = 3.129, *p* = 0.047). Swiss mice of both sexes exposed to stress for 8 weeks showed a shorter latency to immobility (*p* = 0.002, Figure 4C), but not at 6 (*p* = 0.702, Figure 4B) or 4 weeks (*p* = 0.370, Figure 4A) of exposure. For the total immobility time (Figure 4D–F), a significant intervention effect was detected (F_(1,123)_ = 4.845, *p* = 0.030), showing that CUMS‐exposure influenced forced swimming response regardless of sex or stress duration. For this outcome, a significant sex vs. stress duration interaction was also detected (F_(2,123)_ = 13.289, *p* < 0.001). Post hoc analysis revealed a sex difference in total immobility time, with males exhibiting greater immobility than females after 4 (*p* < 0.001) and 6 weeks (*p* < 0.001) of stress (Figure 4D,E). However, an inversion of this pattern is observed after 8 weeks of CUMS exposure (Figure 4F): in this cohort, females showed higher immobility time than males (*p* = 0.003). Most importantly, while male mice's immobility remained stable across the different stress exposure periods (*p* > 0.147), females presented a significant increase in total immobility time after 8 weeks of CUMS, compared to the 4‐ (*p* < 0.001) and 6‐week intervals (*p* < 0.001). Taken together, this profile of stress‐induced changes in immobility indicates that stress‐coping‐related changes were observed mostly in females but also in male mice, and became more evident after 8 weeks of stress exposure.

### 3.5 CUMS Led to an Increase in Serum Corticosterone in Female but Not Male Mice

**FIGURE 5:**
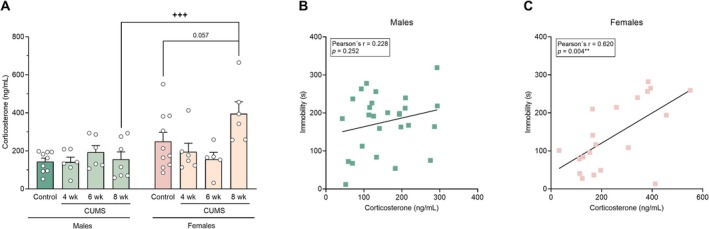
Eight weeks of CUMS exposure increased serum corticosterone in females. (A) Corticosterone plasma levels (ng/mL) of male and female mice exposed to CUMS. Control groups include 2–3 animals from each experimental cohort (*n* = 4–10). Two‐way ANOVA, interaction factor, ^**+++**^ *p* < 0.001 in the post‐test. (B) Correlation between corticosterone plasma levels and forced swimming immobility time in male Swiss mice exposed or not to CUMS (*n* = 27). (C) Correlation between corticosterone plasma levels and forced swimming immobility time in female Swiss mice exposed or not to CUMS (*n* = 20). Pearson's correlation test, **p = 0.004.

Finally, chronic stress exposure alters the HPA axis, leading to increased serum corticosterone levels (Palumbo et al. 2020). This effect was also observed in our CUMS model, but only in female mice (Figure 5). In general, female mice exhibited higher corticosterone levels than males (sex factor, F_(1,46)_ = 7.612, *p* = 0.008). Also, a significant sex and intervention interaction was detected (F_(3,46)_ = 3.823, *p* = 0.016). Eight weeks of stress exposure increased corticosterone serum levels in female mice, reaching a trend level for statistical significance compared to control females (*p* = 0.057) and significantly different from 8‐week stressed male mice (*p* < 0.001) in post hoc tests. In addition, we searched for an association between plasma corticosterone levels and the immobility time during the forced swimming test, since these two measures are frequently described as related. We found a moderate positive correlation between these outcomes in females (Pearson's *r* = 0.620, *p* = 0.004), indicating that the higher the immobility during forced swimming, the higher the basal corticosterone levels (Figure 5C). This relationship was not observed in males (Figure 5B, Pearson's *r* = 0.228, *p* = 0.252). Thus, our data point to a sex‐dependent effect of CUMS exposure on HPA axis function.

## 4 Discussion

This study explored the effects of CUMS exposure in male and female Swiss mice, comparing behavioral and serum corticosterone changes usually associated with depression‐like and high‐anxiety phenotypes. In summary, our results showed that 8 weeks of CUMS exposure were needed to induce robust changes in behaviors related to depressive‐like symptomatology in the Swiss outbred mouse strain, observed by an increased immobility time and decreased latency to immobility in the forced swimming test. However, this phenotype was correlated with increased serum corticosterone levels only in females. Moreover, CUMS exposure led to sex‐dependent effects in anxiety‐related behaviors: while females developed increased anxiety after 4 weeks of stress exposure, males showed an initial increase followed by a behavioral shift consistent with a differential stress adaptation strategy. Also, a mild increase in social preference was induced by stress exposure. No stress‐related effects were observed on weight gain, locomotion, or sucrose preference.

The CUMS model has been extensively described as inducing behavioral changes associated with depression and anxiety in rodents. Because of its high translational validity, it is a powerful tool to elucidate the role of sex in the neurophysiology of stress‐related disorders, such as major depression and generalized anxiety (Franceschelli et al. 2014). Since its first description by Paul Willner et al. (1987), one of the main bases of the CUMS model is the manifestation of anhedonia, the reduced ability to experience pleasure. Therefore, the SPT has become a hallmark of successful model implementation (Liu et al. 2018). In our experimental conditions, however, we did not detect anhedonia in any of our experimental groups. Willner (2017), while revisiting the model and its adaptation to mice, described how individual differences can affect the susceptibility and reproducibility of the CUMS model, including inter and intraspecific factors, genomic manipulations, and natural variation. Swiss mice have already been described as a resistant strain for the development of anhedonia in this model (Yalcin et al. 2008). A similar pattern of response to CUMS in other behavioral tasks, without changes in sucrose preference, has already been described in other outbred rodent strains such as Sprague–Dawley rats (Chang and Grace 2014). A meta‐analysis of sucrose preference deficits induced by CUMS (Antoniuk et al. 2019) reported a limited number of studies using Swiss mice, limiting the analysis of publication bias. As a result, the sample of published papers may not be representative of all studies conducted, due to the difficulty in observing CUMS‐induced anhedonia in this strain. However, in our work, the SPT was conducted in a short‐duration session (30 min) with a 2.5% sucrose solution, which differs from most protocols that use longer testing periods and lower sucrose concentrations (e.g., 1%). Although SPT protocols lack full standardization and different methodological approaches have been described (Primo et al. 2023), differences in methodological parameters may influence the outcomes and limit direct comparability across studies. Indeed, the test's sensitivity in detecting anhedonia following stress exposure, as well as the animal's motivation to consume sucrose, are known to vary with both the duration of the assessment and the sucrose concentration (Scheggi et al. 2018). Therefore, the present findings should be interpreted within the context of these methodological constraints.

Chronic stress has often been associated with reduced body weight, commonly interpreted as a consequence of decreased food intake due to anhedonia and diminished motivation for palatable rewards (Maniam and Morris 2012). In this context, the absence of anhedonia‐like behavior in the present study may contribute to the lack of significant stress‐induced changes in the Swiss mice's body weight. Nevertheless, increases (Tran and Gellner 2023) or no changes (Neves and Grace 2019) in body weight have also been reported depending on the nature, duration, and intensity of the stressors. Additionally, strain‐specific characteristics may contribute to variability in physiological responses to chronic stress. Thus, the lack of weight change can be interpreted as an indicator of this strain's high resilience to stress.

Despite the lack of anhedonia, some notable sex‐dependent CUMS‐induced changes were observed in other behavioral tasks. A mild increase in social preference was induced by stress in both males and females. Despite the general assumption that chronic stress impairs social behavior (Toth and Neumann 2013), more recent studies have demonstrated that different types of stress do not influence this behavioral outcome (Rincón‐Cortés and Grace 2022; Guimarães et al. 2023). Thus, social impairment seems to be more robustly induced by models with a strong social component, such as social defeat or social fear conditioning (Golden et al. 2011; Xu 2026). Moreover, despite the statistical significance, the observed increase in social preference induced by CUMS in our work is minor and may not have any meaningful biological impact on mice's social behavior. Thus, we can assume that social behavior is preserved in Swiss mice exposed to CUMS.

Analysis of the thigmotaxis‐related parameters in the OF revealed distinct temporal patterns of behavioral changes between males and females. Usually, the exploration of the center of the OF has a positive correlation with the time spent in the open arms of the elevated plus maze, a task classically used to assess anxious phenotype (Carola et al. 2002). Both males and females showed a decrease in field exploration time after 4 weeks of stress. A significant reduction in distance travelled at the OF center was observed, which was not accompanied by a significant reduction in center spent time, even though the average time for CUMS groups is lower than that of controls. This can be interpreted in two different ways: animals are either moving slowly towards the center, which could indicate a risk assessment strategy (Quartermain et al. 1996); or animals are stopping at the center zone and doing different actions that do not account for distance, such as freezing or exploring the center vertically. In all situations, the most likely explanation is a stress‐induced anxiogenic effect of CUMS in the OF exploratory profile. However, CUMS‐exposed females only presented this augmented anxiety‐like behavior in the OF after 4 weeks of stress, while no changes were observed after 6 and 8 weeks, indicating an adaptation to the anxiogenic effects of CUMS. By contrast, males showed an inversion in their stress response: from a mild increase in anxiety in the 4‐week cohort to an increase in field center exploration after longer periods of stress exposure. These data indicate that male mice use a different habituation strategy for maladaptive stress responses in this task than females.

Results of the forced swimming test and corticosterone levels further corroborate a sex difference in stress response. Initially introduced in mice by Porsolt et al. (1977) for screening potential antidepressant drugs, the forced swimming test is commonly used as a behavioral paradigm to investigate stress‐coping responses and as part of a repertoire of behavioral changes associated with a depressive‐like phenotype. Despite huge discussion, the immobility during forced swimming can be interpreted as a reduction in the persistence of escape‐directed behavior, that is, a measure of behavioral despair. Additionally, swimming immobility is associated with increased sensitivity to stressful stimuli and with the acquisition of a passive coping response (Cryan 2010; Chen et al. 2015). In this test, our study identified a sex‐independent increase in immobility time following CUMS exposure. However, the effect size observed in females is greater than that observed in males, demonstrating females' greater sensitivity to stress. Moreover, there is a correlation between corticosterone levels and forced swimming immobility, which has been extensively described in both rats (Abel 1993; Báez and Volosin 1994) and mice (Zhao et al. 2008). Our data showed that serum corticosterone was moderately correlated with immobility time in females but not in males, as previously described for rats (Dalla et al. 2005). Therefore, our results are consistent with clinical data showing that women are more prone to stress‐induced detrimental outcomes than men.

In our experimental conditions, changes in behaviors related to depressive‐like symptomatology were robustly induced in Swiss mice only after 8 weeks of CUMS exposure. This length of stress exposure is longer than what is usually required to induce the phenotype in rats (Chang and Grace 2014; Neves and Grace 2019) or isogenic mouse strains, especially the C57BL/6, which appears to develop an adaptation to stress after such long periods of exposure (Pałucha‐Poniewiera et al. 2020). However, most published studies using mice employ foot shock or cold exposure, which are not necessarily mild stressors, or even use swimming as a stressor and behavioral endpoint, introducing a memory bias in their results (Monteiro et al. 2015; Mehta et al. 2017). Our data suggest that Swiss mice can be considered a strain with high stress resilience, requiring a protocol with more aggressive stress duration and intensity patterns to achieve a robust phenotype. Given that the general population presents high variability in patterns of stress resilience and sensitivity, outbred mouse strains such as the Swiss might be more closely replicating this characteristic, highlighting the importance of also using outbred strains to study the pathophysiology of these disorders.

Although chronic stress is a risk factor for both depressive and anxiety disorders (Mir and Rivarola 2022), anxiogenic effects are more directly associated with shorter exposure times, while the depressive phenotype is associated with more prolonged stress exposure (Belujon and Grace 2015). Our data demonstrated such a pattern: behaviors related to high anxiety were observed as early as 4 weeks of CUMS exposure, while behaviors correlated with a depressive symptomatology appeared only after 8 weeks. Thus, our results corroborate both preclinical and clinical findings, confirming the translational validity of the model under our experimental conditions.

Finally, we identified some innate behavioral sex differences in our study. Male mice showed a higher sucrose preference than females in all cohorts. In this regard, the literature shows conflicting evidence regarding differences between males and females in sucrose preference. Michailidis et al. (2021) also identified a higher sucrose preference in males than in females using a Swiss‐derived outbred mouse strain (CD1). However, most studies demonstrate a more pronounced sucrose preference in females (Dalla et al. 2005; Kamper et al. 2009). Hormonal differences and environmental factors can differentially affect hedonic brain circuits (Dalla et al. 2005). The interest in sweet taste appears to be partially influenced by estradiol levels (Clarke and Ossenkopp 1998), which may help explain the sex‐related difference observed in this behavioral task. Basal sex differences were also identified in the response to an acute stressful situation in the forced swimming test. After 4 and 6 weeks of CUMS, males showed longer immobility times than females. This finding may be related to differences in body mass between the sexes, with females being lighter. Animals with greater weight or older age tend to display increased immobility, potentially reflecting reduced motor performance, as body weight is known to influence behavioral outcomes in the forced swimming test (Brenes et al. 2008; Bogdanova et al. 2013). Conversely, in the group subjected to 8 weeks of CUMS, females showed longer immobility time compared to males. However, this increase in females' immobility time seems to be due to their higher stress sensitivity.

Finally, CUMS is recognized as one of the stress models most associated with changes in the female estrous cycle (Poitras et al. 2024). Thus, possible stress interference with females' cyclicity was investigated by weekly cycle monitoring in both control and CUMS‐exposed females. Our data showed that all stages of the estrous cycle were observed in CUMS‐exposed mice regardless of stress duration, supporting continuous cycling throughout the study. The idea that the estrous cycle is a relevant independent variable in experiments with female subjects has been recently rejected (Prendergast et al. 2014). However, we cannot discard the possible interference of weekly vaginal wash as an additional stressor imposed on females but not males in our experimental design. Thus, the weekly estrous checking can be considered a confounding factor in the sex differences observed in our study.

## 5 Conclusion

In summary, our data suggest that females are more sensitive to stress‐induced maladaptive behavioral responses than males, showing a stress‐induced anxiogenic effect, longer latency and higher swimming immobility, and a stress‐induced rise in corticosterone levels. In addition, Swiss mice can be considered an outbred mouse strain highly resilient to stress, since they require longer exposure to present behaviors related to depressive‐like symptomatology than is typically observed in other strains. Taken together, these assumptions confirm the high translational value of using outbred strains to study the pathophysiology of stress‐related neuropsychiatric disorders and the mechanisms of stress resilience.

## Author Contributions

**Rachel de Barros‐Telles:** methodology, data curation, investigation, formal analysis, visualization, writing – original draft. **Ana Clara F. da Silva:** methodology, data curation, investigation, formal analysis, visualization, writing – original draft. **Isabelle Medeiros:** data curation, investigation, formal analysis. **Renata V. de Souza:** data curation, investigation, formal analysis. **Aline R. Cardoso:** data curation, investigation, formal analysis, writing – review and editing. **Isis N. O. Souza:** data curation, investigation, formal analysis, visualization, writing – review and editing. **Gilda A. Neves:** conceptualization, methodology, investigation, formal analysis, visualization, resources, supervision, project administration, funding acquisition, writing – review and editing, writing – original draft.

## Funding

This work was supported by the Conselho Nacional de Desenvolvimento Científico e Tecnológico, the Fundação Carlos Chagas Filho de Amparo à Pesquisa do Estado do Rio de Janeiro (E‐26/211.001/2019), and the Coordenação de Aperfeiçoamento de Pessoal de Nível Superior.

## Ethics Statement

All manipulation was performed according to the ARRIVE guidelines, the Council Directive 2010/63EU of the European Parliament, the Council of 22 September 2010 on the protection of animals used for scientific purposes, and Brazilian Guidelines (Institutional Ethics Committee Approval no. 100/19).

## Conflicts of Interest

The authors declare no conflicts of interest.

## Supporting information


**Table S1:** Estrous cycle phase determination of female mice exposed to CUMS and control.
**Table S2:** Three‐way repeated measures ANOVA results of data from Figure 1 (weight gain).
**Table S3:** Three‐way ANOVA results of data from Figure 2 (sucrose preference and social approach tests).
**Table S4:** One‐sample Student t test results of data from Figure 2 (sucrose preference and social approach tests). Reference value = 50%.
**Table S5:** Three‐way ANOVA results of data from Figure 3 (open field test).
**Table S6:** Three‐way ANOVA results of data from Figure 4 (forced swimming test).

## Data Availability

The complete dataset of this study is available under the following link: https://doi.org/10.6084/m9.figshare.32101357.
