# Supplementary material for: Sex Differences in Behavioral Responses to Chronic Unpredictable Mild Stress in Swiss Mice
Source: Eur J Neurosci. 2026 Jul 16;64(2):e70631. doi: 10.1111/ejn.70631 (PMC13373618; doi:10.1111/ejn.70631)
Supplement: Supplementary file 1 — Table S1: Estrous cycle phase determination of female mice exposed to CUMS and control. Table S2: Three‐way repeated measures ANOVA results of data from Figure 1 (weight gain). Table S3: Three‐way ANOVA results of data from Figure 2 (sucrose preference and social approach tests). Table S4: One‐sample Student t test results of data from Figure 2 (sucrose preference and social approach tests). Reference value = 50%. Table S5: Three‐way ANOVA results of data from Figure 3 (open field test). Table S6: Three‐way ANOVA results of data from Figure 4 (forced swimming test). [file EJN-64-0-s001.pdf]

Sex differences in behavioral responses to chronic unpredictable mild stress in Swiss mice

Rachel de Barros-Telles[1,*], Ana Clara F. da Silva[1,*], Isabelle Medeiros[1], Renata V. de Souza[1], Aline R. Cardoso[1], Isis N. O. Souza[1], Gilda A. Neves[1]

1 - Laboratory of Molecular Pharmacology, Institute of Biomedical Sciences, Universidade Federal do Rio de Janeiro, Brazil

*Both authors contributed equally

Supporting Information

Baseline sucrose preference test

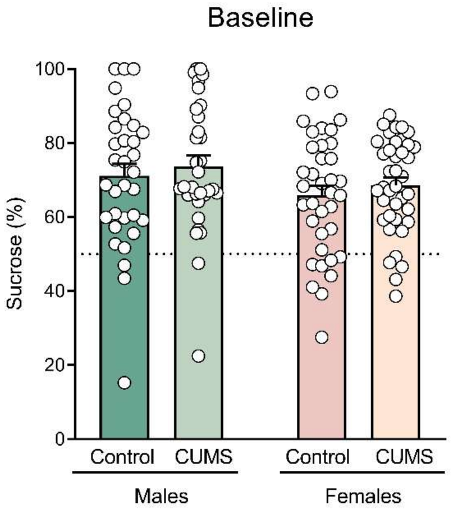

Figure S1. Baseline sucrose preference was equivalent in males and females randomized to CUMS exposure or control group (n = 32-39). Two-way ANOVA, sex factor: $F_{(1,139)} = 3.476$, $p = 0.064$, intervention factor: $F_{(1,139)} = 0.913$, $p = 0.341$, interaction: $F_{(1,139)} = 0.00276$, $p = 0.958$. One-sample Student's t-test. Reference value: 50%. Males: control, $t_{(32)} = 6.526$, $p < 0.001$; CUMS, $t_{(31)} = 7.621$, $p < 0.001$. Females: $t_{(35)} = 5.857$, $p < 0.001$; CUMS $t_{(38)} = 9.137$, $p < 0.001$. Results expressed as mean ± S.E.M.

Estrous cycle analysis

Females were evaluated regarding their estrous cycle phase at baseline and then weekly during stress exposure. Briefly, mice were immobilized, and the vaginal opening was washed with 100 uL of sterile saline solution at room temperature. Vaginal washes were immediately added to histological slides and checked under a light microscope by a blind experimenter. Results can be found in Table S1.

Table S1. Estrous cycle phase determination of female mice exposed to CUMS and control.

Animal	n	Proestrus n (%)	Estrus n (%)	Metestrus n (%)	Diestrus n (%)	Indetermined n (%)
**Control groups**						
Baseline	36	5 (13.9%)	6 (16.7%)	19 (52.8%)	5 (13.9%)	1 (2.8%)
Week 1	36	2 (5.6%)	4 (11.1%)	18 (50.0%)	11 (30.6%)	1 (2.8%)
Week 2	36	9 (25.0%)	2 (5.6%)	19 (52.8%)	5 (13.9%)	1 (2.8%)
Week 3	36	8 (22.2%)	1 (2.8%)	13 (36.1%)	12 (33.3%)	2 (5.6%)
Week 4	36	4 (11.1%)	3 (8.3%)	12 (33.3%)	13 (36.1%)	4 (11.1%)
Week 5	36	5 (13.9%)	6 (16.7%)	13 (36.1%)	11 (30.6%)	1 (2.8%)
Week 6	24	3 (12.5%)	2 (8.3%)	10 (41.7%)	9 (37.5%)	0 (0.0%)
Week 7	24	8 (33.3%)	1 (4.2%)	4 (16.7%)	9 (37.5%)	2 (8.3%)
Week 8	12	2 (16.7%)	2 (16.7%)	6 (50.0%)	2 (16.7%)	0 (0.0%)
Week 9	12	3 (25.0%)	0 (0.0%)	8 (66.7%)	1 (8.3%)	0 (0.0%)
**CUMS groups**						
Baseline	40	8 (20.0%)	9 (22.5%)	12 (30.0%)	10 (25.0%)	1 (2.5%)
Week 1	40	7 (17.5%)	3 (7.5%)	12 (30.0%)	14 (35.0%)	4 (10.0%)
Week 2	40	12 (30.0%)	2 (5.0%)	14 (35.0%)	12 (30.0%)	0 (0.0%)
Week 3	40	8 (20.0%)	6 (15.0%)	14 (35.0%)	12 (30.0%)	0 (0.0%)
Week 4	40	4 (10.0%)	5 (12.5%)	17 (42.5%)	8 (20.0%)	6 (15.0%)
Week 5	40	9 (22.5%)	3 (7.5%)	19 (47.5%)	8 (20.5%)	1 (2.5%)
Week 6	24	7 (29.2%)	3 (12.5%)	7 (29.2%)	6 (25.0%)	1 (4.2%)
Week 7	24	3 (12.5%)	4 (16.7%)	6 (25.0%)	9 (37.5%)	2 (8.3%)
Week 8	12	0 (0.0%)	4 (33.3%)	6 (50.0%)	2 (16.7%)	0 (0.0%)
Week 9	12	3 (25.0%)	4 (33.3%)	5 (41.7%)	0 (0.0%)	0 (0.0%)

Statistical analysis

Table S2. Three-way repeated measures ANOVA results of data from Figure 1 (weight gain).

Factor	Weigh gain (g)
Sex factor	$F_{(1,44)} = 95.285$<br>$p < 0.001$***
Intervention factor	$F_{(1,44)} = 0.115$<br>$p = 0.736$
Sex x intervention	$F_{(1,44)} = 0.498$<br>$p = 0.484$
Time factor (weeks)	$F_{(8,352)} = 19.244$<br>$p < 0.001$***
Sex x time	$F_{(8,352)} = 28.818$<br>$p < 0.001$***
Intervention x time	$F_{(8,352)} = 2.290$<br>$p = 0.016$*
Triple interaction	$F_{(8,352)} = 0.841$<br>$p = 0.567$

n.a. – not applicable

Table S3. Three-way ANOVA results of data from Figure 2 (sucrose preference and social approach tests).

Factor	Sucrose Preference (%)	Social Preference (%)
Sex factor	$F_{(1,129)} = 21.658$<br>$p < 0.001$***	$F_{(1,132)} = 2.989$<br>$p = 0.086$
Intervention factor	$F_{(1,129)} = 2.347$<br>$p = 0.128$	$F_{(1,132)} = 4.206$<br>$p = 0.042$*
Stress duration factor	$F_{(2,129)} = 6.360$<br>$p = 0.002$**	$F_{(2,132)} = 0.0346$<br>$p = 0.966$
Sex x intervention	$F_{(1,129)} = 1.609$<br>$p = 0.207$	$F_{(1,132)} = 0.198$<br>$p = 0.657$
Sex x stress duration	$F_{(2,129)} = 0.0079$<br>$p = 0.992$	$F_{(2,132)} = 4.887$<br>$p = 0.009$**
Intervention x stress duration	$F_{(2,129)} = 0.380$	$F_{(2,132)} = 0.878$

	p = 0.684	p = 0.418
Triple interaction	$F_{(2,129)}$ = 0.613<br>p = 0.543	$F_{(2,132)}$ = 0.464<br>p = 0.630

Table S4. One-sample Student t test results of data from Figure 2 (sucrose preference and social approach tests). Reference value = 50%.

**Data**			**Sucrose Preference (%)**	**Social Preference (%)**
4 weeks	Males	Control	$t_{(11)}$ = 14.19<br>p < 0.001***	$t_{(11)}$ = 14.31<br>p < 0.001***
		CUMS	$t_{(10)}$ = 7.769<br>p < 0.001***	$t_{(11)}$ = 15.56<br>p < 0.001***
	Females	Control	$t_{(11)}$ = 6.066<br>p < 0.001***	$t_{(11)}$ = 6.134<br>p < 0.001***
		CUMS	$t_{(12)}$ = 4.5019<br>p < 0.001***	$t_{(15)}$ = 4.801<br>p < 0.001***
6 weeks	Males	Control	$t_{(11)}$ = 10.06<br>p < 0.001***	$t_{(11)}$ = 6.010<br>p < 0.001***
		CUMS	$t_{(10)}$ = 13.66<br>p < 0.001***	$t_{(10)}$ = 11.84<br>p < 0.001***
	Females	Control	$t_{(10)}$ = 5.458<br>p < 0.001***	$t_{(11)}$ = 5.239<br>p < 0.001***
		CUMS	$t_{(11)}$ = 2.216<br>p = 0.049*	$t_{(9)}$ = 7.164<br>p < 0.001***
8 weeks	Males	Control	$t_{(11)}$ = 9.140<br>p < 0.001***	$t_{(11)}$ = 5.512<br>p < 0.001***
		CUMS	$t_{(11)}$ = 15.51<br>p < 0.001***	$t_{(11)}$ = 7.848<br>p < 0.001***
	Females	Control	$t_{(10)}$ = 8.710<br>p < 0.001***	$t_{(11)}$ = 4.043<br>p = 0.002**
		CUMS	$t_{(11)}$ = 8.127<br>p < 0.001***	$t_{(11)}$ = 10.92<br>p < 0.001***

Table S5. Three-way ANOVA results of data from Figure 3 (open field test).

Factor	Total distance traveled (cm)	Distance traveled in the center (%)	Time spent in the center (s)
Sex factor	$F_{(1,132)}$ = 0.00743 *p* = 0.931	$F_{(1,132)}$ = 3.547 *p* = 0.062	$F_{(1,47)}$ = 0.130 *p* = 0.720
Intervention factor	$F_{(1,132)}$ = 0.0132 *p* = 0.909	$F_{(1,132)}$ = 0.00419 *p* = 0.948	$F_{(1,47)}$ = 1.682 *p* = 0.201
Stress duration factor	$F_{(2,132)}$ = 0.368 *p* = 0.693	$F_{(2,132)}$ = 5.153 *p* = 0.007**	$F_{(1,47)}$ = 0.0121 *p* = 0.913
Sex x intervention	$F_{(1,132)}$ = 0.570 *p* = 0.452	$F_{(1,132)}$ = 6.798 *p* = 0.010*	$F_{(1,42)}$ = 0.158 *p* = 0.693
Sex x stress duration	$F_{(2,132)}$ = 0.894 *p* = 0.412	$F_{(2,132)}$ = 3.114 *p* = 0.048*	$F_{(1,42)}$ = 1.269 *p* = 0.266
Intervention x stress duration	$F_{(2,132)}$ = 0.597 *p* = 0.552	$F_{(2,132)}$ = 3.950 *p* = 0.022*	$F_{(1,42)}$ = 5.808 *p* = 0.020*
Triple interaction	$F_{(2,132)}$ =0.350 *p* = 0.705	$F_{(2,132)}$ = 1.843 *p* = 0.162	$F_{(1,43)}$ = 9.954 *p* = 0.003**

Table S6. Three-way ANOVA results of data from Figure 4 (forced swimming test).

Factor	Immobility latency (s)	Immobility duration (s)
Sex factor	$F_{(1,123)}$ = 0.386 *p* = 0.536	$F_{(1,123)}$ = 4.908 *p* = 0.029*
Intervention factor	$F_{(1,123)}$ = 4.385 *p* = 0.038*	$F_{(1,123)}$ = 4.845 *p* = 0.030*
Stress duration factor	$F_{(2,123)}$ = 0.681 *p* = 0.508	$F_{(2,123)}$ = 2.588 *p* = 0.079
Sex x intervention	$F_{(1,123)}$ = 2.827 *p* = 0.095	$F_{(1,123)}$ = 0.285 *p* = 0.595
Sex x stress duration	$F_{(2,123)}$ = 0.389 *p* = 0.678	$F_{(2,123)}$ = 13.289 *p* < 0.001***
Intervention x stress duration	$F_{(2,123)}$ = 3.129 *p* = 0.047*	$F_{(2,123)}$ = 1.620 *p* = 0.202
Triple interaction	$F_{(2,123)}$ = 1.802 *p* = 0.169	$F_{(2,123)}$ = 1.585 *p* = 0.209

Table S7. Two-way ANOVA results of data from Figure 5 (corticosterone assay).

Factor	Corticosterone (ng/mL)
Sex factor	$F_{(1,46)} = 7.612$<br>*p* = 0.008**
Stress duration factor	$F_{(3,46)} = 2.859$<br>*p* = 0.047*
Interaction	$F_{(3,46)} = 3.823$<br>*p* = 0.016*
